# The Construction of a Standard Karyotype of Intermediate Wheatgrass and Its Potential Progenitor Species

**DOI:** 10.3390/plants14020196

**Published:** 2025-01-12

**Authors:** Lin Wang, Shuang Liang, Fei Qi, Yinguang Bao, Richard R.-C. Wang, Xingfeng Li

**Affiliations:** 1 State Key Laboratory of Wheat Improvement, Shandong Agricultural University, Tai’an 271018, China; xixicwan@163.com (L.W.); sliang_sdau@163.com (S.L.); 2021010020@sdau.edu.cn (F.Q.); ygbao@sdau.edu.cn (Y.B.); 2 Tai’an Subcenter of National Wheat Improvement Center, Agronomy College, Shandong Agricultural University, Tai’an 271018, China; 3 USDA-ARS, Forage & Range Research Laboratory (FRRL), Logan, UT 84322-6300, USA

**Keywords:** Triticeae, karyotype analysis, genome constitution, FISH, evolution

## Abstract

The genome composition of intermediate wheatgrass (IWG; *Thinopyrum intermedium* (Host) Barkworth and D.R. Dewey; 2n = 6x = 42) is complex and remains to be a subject of ongoing investigation. This study employed fluorescence in situ hybridization (FISH) to analyze the karyotype of *Th. intermedium* and its related species. With the St_2_-80 probe derived from *Pseudoroegneria strigosa* and the pDb12H probe from *Dasypyrum breviaristatum*, FISH analysis classified the chromosomes of *Th. intermedium* as **J^vs^J^vs^J^r^J^r^StSt**. FISH karyotype was established using pSc119.2-1, (GAA)_10_, AFA-3, AFA-4, pAs1-1, pAs1-3, pAs1-4, and pAs1-6 as a combined multiplex oligonucleotide probe. MATO software was used to analyze chromosome length, arm ratio, and karyotype structure. The karyotype formula of *Th. intermedium* is K(2n) = 6X = 42 = 36m + 6sm, and that of *Th. junceiforme* is K(2n) = 4X = 28 = 22m + 6sm. The karyotype formula of *Th. elongatum* and *Th. bessarabicum* is K(2n) = 2X = 14 = 12m + 2sm, of *Ps. spicata* is K(2n) = 2X = 14 = 2M + 12m, and of *Da. villosum* is K(2n) = 2X = 14 = 12m + 2sm. Based on the results of FISH, standard karyotypes of *Th. intermedium* and its potential progenitor species were constructed. These standard karyotypes revealed that there was evolutionary parallelism between genome and karyotype, but due to the complexity of evolution, the FISH signal of *Th. intermedium* was abundant and asymmetrical.

## 1. Introduction

Intermediate wheatgrass (IWG) is a wild perennial herb with well-developed roots and strong cold-resistance [1,2,3]. It is immune to various diseases, such as powdery mildew, three wheat rust diseases (leaf, stem, and stripe rust), and highly resistant to yellow dwarf virus (BYDV) and wheat streak mosaic virus (WSMV) [4,5,6,7,8,9]. IWG is an important tertiary gene source for wheat genetic improvement [10,11,12,13]. IWG is the first widely used commercial perennial food crop, sold under the trade name “Kernza” [14,15,16,17]. Therefore, as an important forage crop, IWG has attracted the attention of many researchers.

The research on *Th. intermedium* has been abundant, and its genomic symbols have undergone many changes over time. Traditionally, meiotic chromosome pairing in various hybrids was the primary method for identifying the genome composition of the Triticeae species [18,19,20]. Therefore, based on meiotic chromosome pairing and C-band analysis, many different theories have been proposed to explain the genome composition of *Th. intermedium* [18,19,21,22], such as **BEF** and **E1E2X** [23]. Liu and Wang [24,25] proposed that its genome composition should be **J^e^J^e^S**. Subsequent studies confirmed the presence of the **S** (changed to **St** after 1995 [26]) genome in *Th. intermedium*.

With the development of genomic in situ hybridization (GISH) and fluorescence in situ hybridization (FISH), the differentiation of the *Th. intermedium* genome constitution became clear. The **St** genome in *Th. intermedium* was derived from *Pseudoroegneria* [27,28,29,30]. Chen [31] believed that *Th. intermedium* had three ancestor species, namely diploid *Th. bessarabicum, Th. elongatum,* and a *Pseudoroegneria* species, and proposed the genome symbol as **JJ^s^S**, in which, **J** was derived from *Th. bessarabicum*, or diploid *Th. elongatum*, the **S** genome was homologous to the **S** genome of *Ps. strigosa*, while the **J^s^** genome referred to modified **J**- or **E**-type chromosomes distinguished by the presence of the **S**-genome specific sequences close to the centromere. Qi [32] found that *Th. bessarabicum* and *Th. elongatum* only hybridized with 14 identical chromosomes in *Th. intermedium*, suggesting that there was only one **J** genome in IWG. It was generally accepted that there were **St, J**/**E** genomes in IWG, but the origin of **J^vs^** genomes was controversial [33,34]. Research scholars summarized the previous results and concluded that the chromosomes of the **J**^s^ genome were either hybridized by *Ps. spicata* DNA, or they were hybridized by the specific repeat sequence of the **V**-genome of *Da. villosum.* Either way, the **V**-genome was involved in the evolution of *Th. intermedium*. The development of molecular marker technology from DNA sequences has become a new way to understand the genome composition of *Th. intermedium*. Mahelka [34] sequenced the chloroplasts trnL-F and GBSSI and confirmed that *Th. intermedium* was allohexaploid, and GBSSI data showed that *Th. intermedium* was derived from *Pseudoroegneria* (**St**), *Dasypyrum* (**V**), *Taeniatherum* (**Ta**), *Aegilops* (**D**), and *Thinopyrum* (**J/E**). However, the existence of the V-genome in the genome of *Th. intermedium* was still controversial [35]. Kishii found that STS marker amplification of the **V**-genome was missing in *Th. intermedium*, indicating that the **V**-genome of modern tufted wheat did not exist in *Th. intermedium*.

In response to the controversy, researchers found that *Th. intermedium*’s origins may be more complicated [36]. Tang [37] discovered the presence of **R**-group DNA sequences in *Th. intermedium*, and Wang [38] developed EST-SSR primers from recognized ancestral species and inferred the genomes of wild ancestral species of *Th. intermedium* carrying *Dasypyrum* repeat sequence. Later, St_2_-80 and pDb12h were developed as specific probes of the **St** genome and **V** genome, respectively, for use on *Th. intermedium* [39,40,41]. *Th. intermedium* was evenly divided into three genomes. Therefore, **J^s^** was changed to **J^vs^** and **J** was changed to **J^r^**, and **J^r^J^r^J^vs^J^vs^StSt** was proposed as the genome symbol of *Th. intermedium*. This genome symbol designation has been accepted by many researchers [38,42]. Yang [40] reported that pDb12H could detect all the chromosomes of *Da. breviaristatum*. The pDb12H can only be weakly hybridized with the pericentric region of the eight chromosomes of *Da. villosum*, and the **J^vs^** genome can be found to hybridize with the genomic DNA of *Da. villosum* or the oligonucleotide probe pDb12H, which proves that *Dasypyrum* is the ancestor of Th. *intermedium* [32].

Chromosomes, as carriers of genetic information, are conserved. During the metaphase of mitosis, they become highly condensed, and their structural arrangement at this stage is referred to as the karyotype. A karyotype is not only a taxonomic feature, but also plays an important role in the analysis of related species and the identification of evolutionary patterns of species. Cytological karyotype analysis is one of the means to study the relationships and evolution of a group of species. The apparent characteristics such as chromosome number, shape, size, and centromeric position in the metaphase stage of mitosis can be analyzed, and a series of parameters such as arm ratio, karyotype asymmetry index [43,44], and chromosome length ratio can be calculated to study the trend of karyotype evolution and analyze the evolutionary model of species [45].

It was generally believed that during the growth and development of species, chromosome number, size, centromere location, and other traits were relatively stable, but with the evolution of species, the shape of chromosomes will change greatly, becoming more and more asymmetric, i.e., in the process of species evolution, the trend of karyotype change is from symmetric to asymmetric [45].

In this study, two IWG accessions and the progenitors (diploid and tetraploid) related to IWG were studied using double oligonucleotide fluorescence in situ hybridization, and their karyotypes were analyzed. The aim was to construct standard karyotypes of IWG, and its ancestral diploid and tetraploid species based on the oFISH results.

## 2. Results

### 2.1. Oligo-FISH of IWG and Its Ancestral Diploid Species

In this study, chromosomes of six species (Table 1) were probed with two oligonucleotides, pDb12H and St_2_-80, to distinguish the **J^r^**, **J^vs^**, and **St** genomes. Then, the same chromosome spreads were probed with bulked oligonucleotides consisting of pSc119.2-1, (GAA)_10_, AFA-3, AFA-4, pAs1-1, Pas1-3, pAs1-4, and pAs1-6 after the slides were cleaned of the pDb12H and St_2_-80 probes.

The Oligo-FISH (oFISH) results were the same as in previous studies [32]. Each pattern of the chromosome was plotted; the **J^vs^** genome had the richest fluorescence signal, followed by the **J^r^** genome, and finally the **St** genome, and the signal of the **St** genome was at the distal ends of chromosomes. The signals had different intensities at different sites on the chromosome, and there were blue interstitial regions on the chromosome arms. The results were consistent with previous articles published by Qi [32]. However, the multiplex oligonucleotide FISH patterns revealed variable signals on some chromosomes of all genomes among accessions, or even within an accession.

### 2.2. Karyotype Analysis of Th. intermedium

**Figure 1:**
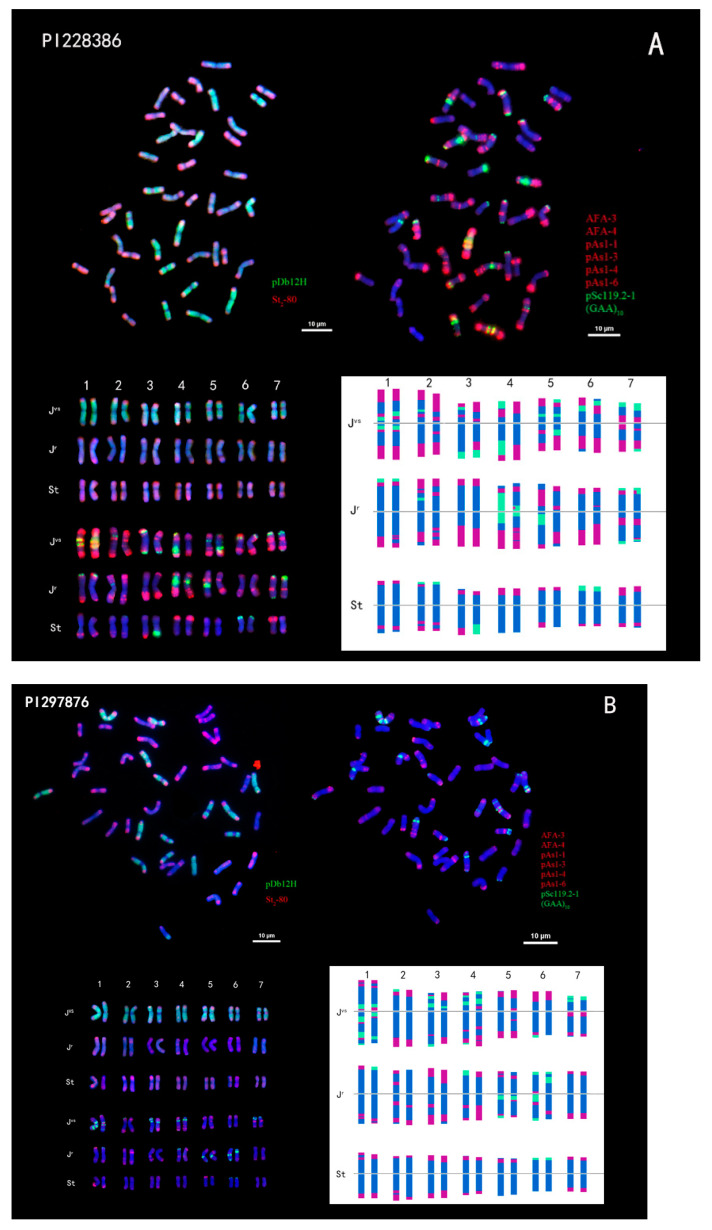
FISH results and karyotypes of *Thinopyrum intermedium* PI 228386 (**A**) and PI 297876 (**B**). Upper left side: probed with pDb12H (green) and St_2_-80 (red). Upper right side: probed with multiplex oligonucleotides, including pSc119.2-1, (GAA)_10_, AFA-3, AFA-4, pAs1-1, Pas1-3, pAs1-4, and pAs1-6. Oligonucleotide multiplex FISH ideogram (bottom). Scale bar: 10 µm.

In PI 228386, there were eight pairs of chromosomes with great differences in the oFISH signal, four pairs of chromosomes with little difference, and the rest of the chromosome signals were almost the same (Figure 1A). For example, for the fourth pair of chromosomes of the **J^vs^** genome, one had an obvious green signal on the long arm end, but the other one did not; the fifth pair of chromosomes of the **J^r^** genome also had a great difference in green signal; and the third pair of chromosomes of the **St** genome had a green signal on one long arm, and only a red signal on the other. Only seven pairs of chromosomes in PI 297876 had great differences in oFISH signals, while the four pairs of chromosomes had little differences (Figure 1B). For example, the red and green signals on the ends of the third and fourth pairs of the **J^vs^** genome were different, the first and fourth pairs of the **J^r^** genome had only a red signal on one end and a green signal on the other end, the green signal distribution of the fifth pair of chromosomes was different, and there was almost no difference between the homologous chromosomes of the **St** genome’s chromosome signals.

In this study, karyotyping was performed using the MATO software [46]. Chromosomal relative lengths, arm ratios, and types are presented in Table 2, Table 3 and Table 4. The chromosome number of *Th. intermedium* was 2n = 6X = 42. The average absolute length of chromosomes in the **St** genome was the shortest; the length of chromosomes in the **J^vs^** genome was similar to that in the **J^r^** genome, but the average absolute length of chromosomes in the **J^r^** genome was slightly longer. The longest chromosome of all chromosomes was in the **J^vs^** genome, and the shortest chromosome was in the **St** genome. The relative chromosome lengths of PI 228386 and PI 297876 ranged from 5.52% to 6.11% and 3.62% to 6.35%, while the ratios of the longest and shortest chromosomes were 1.89 and 1.95, respectively. The arm ratios in PI 228386 and PI 297876 were between 1 and 2. The karyotype formulas of PI 228386 and PI 297876 were K(2n) = 6X = 42 = 36m + 6s. The karyotype asymmetry index of three genomes in PI 228386 and PI 297876 was 57.49%, 54.84%, 56.17%, and 59.60%, 53.99%, 56.52%, respectively (Table 2).

### 2.3. Karyotype Analysis of Tetraploid Species of Thinopyrum

**Figure 2:**
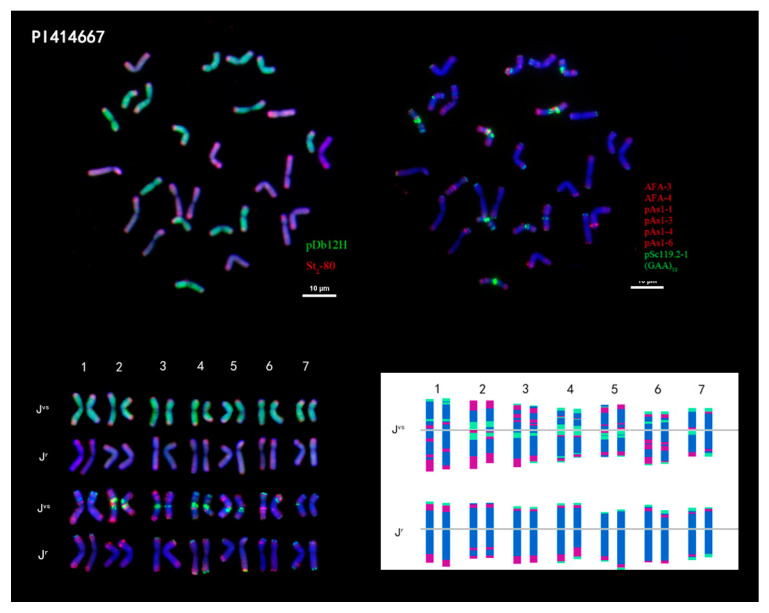
FISH results and karyotypes of *Thinopyrum junceiforme* PI 414667. Upper left side: probed with pDb12H (green) and St2-80 (red). Upper right side: probed with multiplex oligonucleotides, including pSc119.2-1, (GAA)_10_, AFA-3, AFA-4, pAs1-1, Pas1-3, pAs1-4, and pAs1-6. Oligonucleotide multiplex FISH ideogram (bottom). Scale bar: 10 µm.

In PI 414667, only the signal of the seventh pairs of the **J^vs^** genome was very different, the strength of the red signal of the long arm of the sixth pair of chromosomes was different, and moreover, the signal of the other homologous chromosomes was consistent (Figure 2).

The chromosome number of *Th. junceiforme* is 2n = 4X = 28. There were only two genomes of **J^vs^** and **J^r^**, and the average absolute length of chromosomes of the **J^r^** genome was longer than the **J^vs^** genome, which was consistent with the result of *Th. intermedium*. The longest chromosome of all chromosomes was in the **J^r^** genome and the shortest chromosome was in the **J^vs^** genome. The relative chromosome lengths of *Th. junceiforme* ranged from 5.43% to 8.21%, and the ratio of the longest and shortest chromosomes was 1.60. The arm ratio of *Th. junceiforme* was between 1 and 2. The karyotype formulas of *Th. junceiforme* is K(2n) = 4X = 28 = 22m + 6sm. The karyotype asymmetry index of the two genomes in PI 4414667 was 56.99% and 59.29% (Table 3).

### 2.4. Karyotype Analysis of Three Diploid Species That Were Implicated as Progenitors of IWG

**Figure 3:**
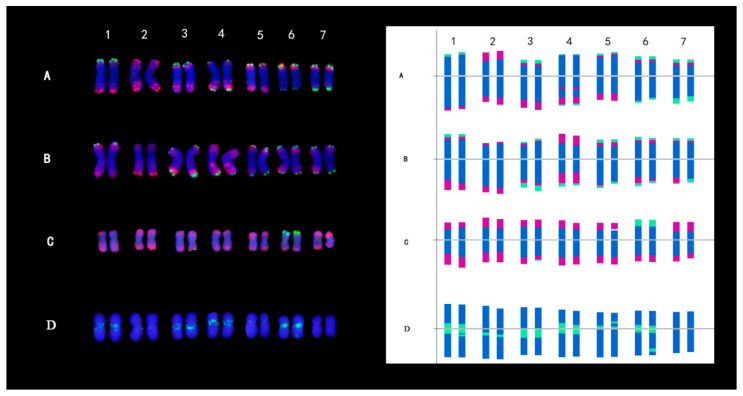
FISH results and karyotypes *of Thinopyrum bessarabicum* (**A**), *Th. Elongatum* (**B**), *Pseudoroegneria spicata* (**C**) and *Dasypyrum villosum* (**D**). Left side: probed with multiplex oligonucleotides, including pSc119.2-1, (GAA)_10_, AFA-3, AFA-4, pAs1-1, Pas1-3, pAs1-4, and pAs1-6. Right side: oligonucleotide multiplex FISH ideogram. Scale bar: 10 µm.

The oFISH signals of *Th. elongatum* and *Th. bessarabicum* were consistent on homologous chromosomes, both located at the ends of chromosome arms (Figure 3A,B). The red and green signals were abundant, and most of the red and green signals existed in the same part of chromosomes at the same time, and the signal similarity between the two species was high. In *Ps. spicata,* only the signals of the seventh homologous chromosome were different at the end of the short arm, in which one chromosome showed a green signal (Figure 3C). In addition, the signals of the other homologous chromosomes were consistent, and the signals were all located at the end of the chromosome. In the oFISH results of *Da. villosum*, there were only green signals, and the homologous chromosomes had the same signals, all located near the centromere. It was worth noting that one pair of chromosomes had no signals (Figure 3D).

According to the previously published results, it can be seen that both *Th. elongatum* and *Th. bessarabicum* were **J^r^** genome, *Ps. spicata* was **St** genome, and *Da. villosum* was **V** genome (Table 4). The relative chromosome lengths of *Th. elongatum* and *Th. bessarabicum* were from 12.42% to 16.58% and 13.02% to 16.29%, and the ratios of the longest and shortest chromosomes were 1.25 and 1.34, respectively. The karyotype formulas for both *Th. elongatum* and *Th. bessarabicum* were K(2n) = 2X = 14 = 12m + 2sm. The relative chromosome lengths of *Ps. spicata* were from 12.78% to 14.88%, and the ratio of the longest and shortest chromosomes of *Ps. spicata* was 1.17. The karyotype formula of *Ps. spicata* was K(2n) = 2X = 14 = 2M + 12m. The relative chromosome lengths of *Da. villosum* were from 12.31% to 16.24%, the ratio of the longest and shortest chromosomes of *Da. villosum* was 1.34. The karyotype formulas of *Da. villosum* were K(2n) = 2X = 14 = 12m + 2sm. The karyotype asymmetry indexes of *Th. elongatum, Th. Bessarabicum*, *Ps. spicata,* and *Da. villosum.* genomes were 57.40%, 58.32%, 55.09%, and 58.62%, respectively.

### 2.5. Karyotype Evolution Analysis of Different Species

**Figure 4:**
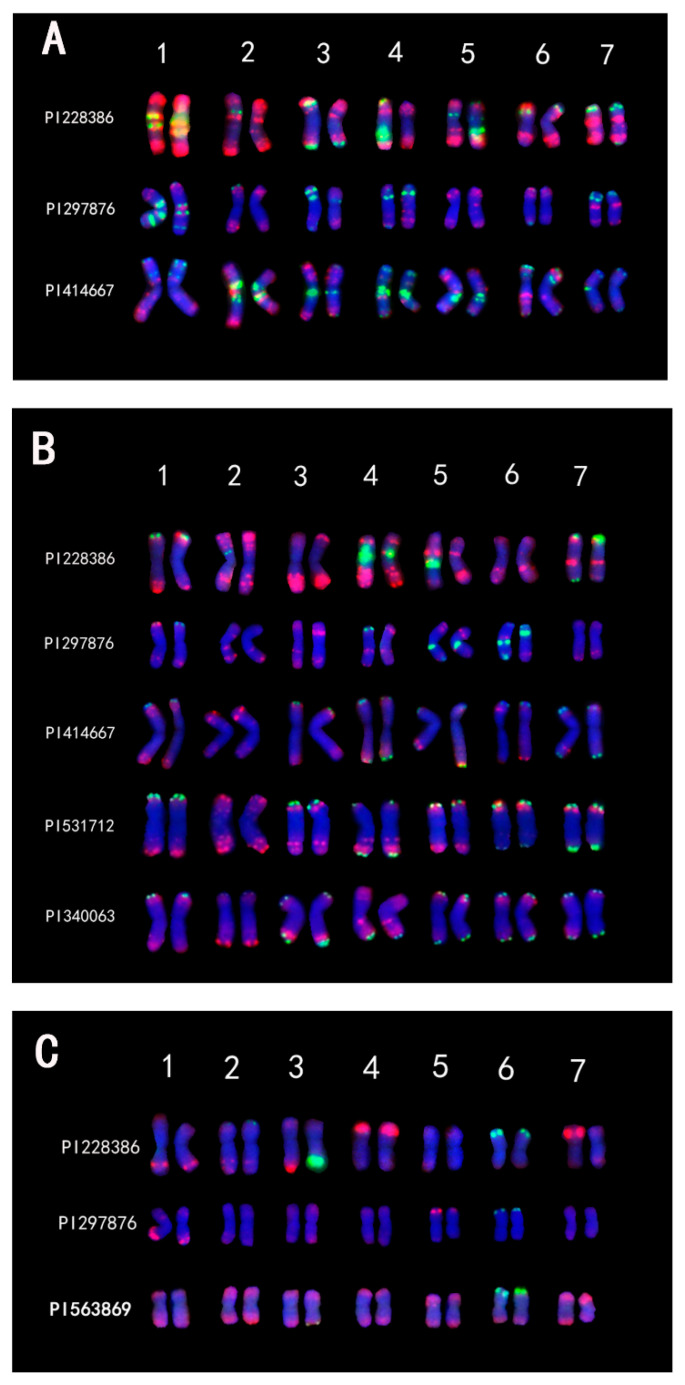
Comparison of karyotypes from the **J^vs^** (**A**), **J^r^** (**B**), and **St** (**C**) genomes.

*Th. intermedium* was compared with the potential tetraploid and diploid progenitor species with known genomes. Comparing the oFISH results of **J^vs^** genomes of the three materials, the signals between them were similar (Figure 4A and Figure S1A). For example, the first pair of chromosomes of PI 228386 and PI 297876 were similar to the second pair of chromosomes of PI 414667, and the sixth pair of chromosomes of PI 228386 was similar to the sixth pair of chromosomes of PI 414667. When the oFISH results of the five **J^r^**-genomes containing materials were analyzed together, it was surprising to find that the results of PI 414667 were very similar to the results of the two diploid materials, but the signals of the two *Th. intermedium* were significantly different (Figure 4B and Figure S1B). Finally, the **St** genome signals of the three materials were compared, which was the group with the most similar signals. In PI 228386, four pairs of chromosome signals were similar to *Ps. spicata*, and in PI 297876, six pairs of chromosome signals were similar, and the other pairs of chromosome signals were also less different (Figure 4C and Figure S1C).

The karyotypes of the same genome of *Th. intermedium* showed high similarity, like the oFISH signal. The karyotype of the same genome in different species is conserved to some extent, with the **J^r^** genome having the most conserved signals.

The **J^vs^** and **St** genomes of IWG exhibited significant differences in chromosome length ratios, average arm ratios, and karyotype asymmetry indices. In contrast, the **J^r^** genome showed moderate differences, likely reflecting the complexity of IWG evolution, while maintaining a consistent overall trend (Table 5).

## 3. Discussion

### 3.1. Progress in the Study of Th. intermedium and Related Species

As an allohexaploid plant, the genome composition of *Th. intermedium* was complex and controversial. Different scholars have used different ways to classify the chromosomes of *Th. intermedium*. Through hybridization and C-band analysis, *Th. intermedium* was divided into three genomes, **E_1_E_1_E_2_E_2_SS** [23]. Later, *Th. intermedium* was considered to be **J^e^J^e^J^e^J^e^SS** [24,25] (while the symbol **S** was later changed to **St** [26]). With genomic in situ hybridizations becoming a powerful tool for analyzing genome constitution, the potential ancestor species of *Th. intermedium* could be investigated. Wang [38] suggested that the genome symbol of *Th. intermedium* group should be changed to **J^vs^J^r^St**, where **J^vs^** and **J^r^** represent the ancestral genomes of the present **J^b^** genome of *Th. bessarabicum* and the **J^e^** genome of *Th. elongatum*, respectively. Only the **St** genome in *Th. intermedium* was unequivocally attributed to the diploid *Pseudoroegneria* species. In our previous study [32], St_2_-80 and pDb12H were used as probes in FISH, and *Th. intermedium* was divided into three genomes. On this basis, we used the same method to conduct a karyotype analysis of *Th. intermedium* and its potential progenitor species to construct the standard karyotype for those species.

At present, it is hypothesized that *Th. intermedium* was a hybrid product of a diploid *Pseudoroegneria* species with a tetraploid *Thinopyrum* species having the **J^vs^J^r^** genome composition. In the present study, the two *Th. intermedium* accessions had the same evolutionary processes, but their FISH signals had internal and external variability. It was speculated that it might have experienced different hybridization events, i.e., the specific species involved in hybridization were not the same, the number of hybridizations was not the same [34], or the out-crossing characteristics of itself [47].

### 3.2. Karyotype Analysis on Th. intermedium and Related Species

Karyotype analysis plays an important role in distinguishing plant species and studying plant evolution. The combination of clear chromosomal images and karyotype analysis data helps to more intuitively analyze plant chromosome changes and karyotype evolution, making it an effective tool for studying plant evolutionary processes and inter-population relationships.

Hsiao et al. [48] conducted karyotype analysis on 22 diploid species of Triticeae and found that the karyotypes of genomes within the same genus were highly similar. Moreover, the chromosome length and the number, size, and location of the satellite were usually the same, but the arm ratio and relative chromosome length were slightly different. Even though the diploid species in the same genus evolved through chromosomal structural changes, this structural difference did not alter the recognizable similarities in the basic karyotype patterns of each genome.

In our study, fluorescence in situ hybridization and karyotype analysis were carried out on the *Th. intermedium* and its potential diploid and tetraploid donor parents based on Qi [32], and the FISH signal similarity between the same species was higher than that of different species. The FISH signal of *Th. elongatum* and *Th. bessarabicum* were conserved and had high similarity to that in the tetraploid *Th. junceiforme*. A small number of chromosomal FISH signals did not change greatly compared with the **J^r^** genome of *Th. intermedium*. Therefore, the **J^r^** genome of diploid species had a high homology with that of tetraploid species. The fluorescence signal of the **St** genome is mainly concentrated at both ends of the chromosome, and like the **J^r^** genome, the signal was conserved and did not change significantly. Therefore, there was no doubt that the **St** genome of *Th. intermedium* was derived from the primitive *Ps. strigose*. The **J^vs^** genome was the most complex genome and no original diploid donor had been identified, and they were very closely related to each other, which brought great difficulties to related studies. Relatively few genomic data had been published, and it could be speculated that it might be formed in hybrids of several diploid species, so its FISH signal was poorly conserved.

From the karyotype analysis, the chromosome karyotypes of the two *Th. intermedium* were highly similar, the relative length changes of the three genomes were consistent, and the arm ratio was slightly different, as follows: The arm ratio of PI 228386 ranged from 1.06 to 1.96, while the arm ratio of PI 297876 ranged from 1.05 to 1.85, and the arm ratio of two diploid *Thinopyrum* ranged from 1.05 to 2.11 and 1.1 to 2.06. The arm ratio of tetraploid *Th. junceiforme* ranged from 1.03 to 2.11.

### 3.3. Karyotype Evolution on Th. intermedium and Related Species

From the perspective of karyotype analysis, Stebbins [45] proposed that karyotype asymmetry correlates with the specificity and specialization of certain plant organs, reflecting the degree of plant evolution. Chromosome length ratio, arm ratio, and karyotype asymmetry index, etc., can reflect the asymmetry of a karyotype between different species, and the greater the difference between them, the greater the karyotype asymmetry. A higher asymmetry index was thought to indicate higher levels of karyotype heterogeneity. Stebbins’ research also suggested that the trend of karyotype evolution was from symmetry to asymmetry and that in phylogenetic evolution, older plants had symmetrical karyotypes, while derived progeny plants tended to have asymmetrical karyotypes.

Oinuma [49] proposed the existence of evolutionary parallelism between genome and karyotype. In other words, different species with the same genome had similar karyotypes. This hypothesis has been tested in many species of Triticeae. The chromosome length ratio, karyotype asymmetry coefficient, and average arm ratio of the three genomes of *Th. intermedium* was analyzed with their potential diploid tetraploid donor parents, and the results were found to be consistent with the Oinuma conclusion. Karyotype asymmetry analysis indicates that PI 297876 is more evolutionarily advanced than PI 228386. The evolution of the **J^vs^** and **St** genomes in *Th. intermedium* aligns with the symmetry-to-asymmetry trend, whereas the **J^r^** genome exhibits more complex evolutionary patterns. The largest asymmetry coefficients of the three genomes of *Th. intermedium* were found in the **J^vs^** genome, while the smallest were found in the **St** genome. The **St** genome is conserved in the evolution process, and the **J^vs^** genome is more complex in the evolution process.

The haploid formula of *Th. intermedium* proposed at present is **J^vs^J^r^St**, and the tetraploid species *Th. junceiforme* with **J^vs^J^r^** was presumed to be its ancestor. In this study, the karyotype formula of *Th. intermedium* is K(2n) = 6X = 42 = 36m + 6sm. The karyotype formula of *Th. junceiforme* is K(2n) = 4X = 28 = 22m + 6sm, and the karyotype formulas of *Th. elongatum*, *Th. bessarabicum* and *Ps.spicata* are K(2n) = 2X = 14 = 12m + 2sm and K(2n) = 2X = 14 = 2M + 12m. According to these karyotype formulas, *Th. junceiforme, Th. elongatum*, *Th. bessarabicum,* and *Ps. spicata* could be the ancestral species of *Th. intermedium*.

## 4. Materials and Methods

### 4.1. Plant Materials

*Thinopyrum intermedium* (2n = 6x = 42), T*h. junceiforme* (2n = 4x = 28), *Th. elongatum* (2n = 2x = 14, **E^e^E^e^**), *Th. bessarabicum* (2n = 2x = 14, **JJ** or **E^b^E^b^**), and *Pseudoroegneria spicata* (2n = 2x = 14, **StSt**) with the PI numbers were kindly provided by the Germplasm Resource Information Network (GRIN) of United States Department of Agriculture (Table 1). *Dasypyrum villosum* (2n = 2x = 14, **VV**) was obtained from Prof. Xingfeng Li, College of Agronomy, Shandong Agricultural University. All plant materials were maintained through selfing at the Tai’an Subcenter of the National Wheat Improvement Center, Tai’an, China.

### 4.2. Probe Preparation

Two oligonucleotide probes, St_2_-80 [39] and pDb12H [40,41], were used for FISH analysis. pDbH12 could serve as a cytogenetic marker used to trace chromatin from the **V^b^** genome. St_2_-80 is a potential and available FISH marker that can be used to distinguish **St** and other genomes in Triticeae. Fluorescent signals of St_2_-80 were labeled with Texas-red-5-dCTP, while pDb12H and were labeled with fluorescein-12-dUTP using the nick translation method. The oligonucleotides (synthesized by Sangon Biotech, Shanghai, China) pSc119.2-1 and (GAA)_10_ were labeled with 5′-FAM (5-carboxyfluorescein) while AFA-3, AFA-4, pAs1-1, Pas1-3, pAs1-4, and pAs1-6 were labeled with 5′-TAMRA (5-carboxytetramethylrhodamine) as described by Du et al., 2017 [50].

### 4.3. Chromosome Preparation and GISH, FISH Protocol

Fresh root-tip cells from germinating seeds were treated with 1.0 MPa nitrous oxide (N_2_O) for 2 h [51], immersed in 90% glacial acetic acid, and subsequently stored in 70% (*v*/*v*) ethanol. After washing with distilled water, the roots were digested in 2% cellulase and 1% pectolase at 37 °C for 55 min. The digested root sections were washed and then mashed in 100% acetic acid to form a cell suspension. The cell suspension is dropped onto glass slides for chromosome preparation according to the procedure used in Prof. Han’s laboratory [52]. After completing the above steps, dispersed without overlapping chromosomes were found and the slides were subjected to FISH. The probe was coated on each slide and covered with a coverslip, the slides were heated at 100 °C for 5 min and incubated at 55 °C overnight. After hybridization, the slides were washed with 2 × SSC for 5 min and then sealed with DAPI. The procedures of FISH and signal detection were carried out according to the method of Du et al. (2017) [50] and He et al. (2017) [53]. Probe labeling, denaturation, image capture, and data processing were described in Cui et al. (2019) [54]. Images were captured using a NIKON Eclipse Ni-U fluorescence microscope (Tokyo, Japan) and processed with NIS-Elements BR 4.00.12 software.

### 4.4. Karyotype Analysis

Chromosomes were paired according to FISH results, and karyotype analysis was performed in combination with MATO software (V4.3) [46]. Chromosomes were sequenced according to the full-length sequence number, and the model map was drawn based on the fluorescence signals.

## 5. Conclusions

In this study, using two oligonucleotides pDb12H and St_2_-80 as probes in FISH is sufficient to distinguish the three genomes **J^vs^**, **J^r^**, and **St** in intermediate wheatgrass. In addition, according to the results of oFISH, the karyotype and evolution of the chromosomes of *Th. intermedium* and related species were analyzed, and their standard karyotype was constructed.

## Figures and Tables

**Table 1:** Plant materials used in the study.

Species	ID	Chr Number	Origin	Note
*Thinopyrum intermedium* (Host) Barkworth and D. R. Dewey	PI 228386 PI 297876	42 42	Iran Former, Soviet Union	
*Th. junceiforme* (A. and D. Löve) A. Löve	PI 414667	28	Greece	listed as *Thinopyrum junceum* (L.) Á. Löve
*Th. bessarabicum* (Savul. and Rayss) A. Löve	PI 531712	14	Estonia	
*Th. elongatum* (Host) D. R. Dewey	PI 340063	14	Turkey	
*Pseudoroegneria spicata* (Pursh) Á. Löve	PI 563869	14	Oregon, USA	
*Dasypyrum villosum* (L.) *Candargy*		14		From X-F Li’s collection

**Table 2:** Chromosome arm ratio (L/S) and type of *Th. intermedium*.

Species	Genome		Chromosome No.						
			1	2	3	4	5	6	7
228286	**J^vs^**	L + S (%)	17.74	15.41	14.43	14.03	12.79	13.64	11.97
		L/S	1.14	1.07	1.78	1.96	1.22	1.21	1.42
		Type	m	m	sm	sm	m	m	m
	**J^r^**	L + S (%)	15.78	15.29	15.26	14.26	14.18	12.81	12.41
		L/S	1.07	1.05	1.08	1.35	1.36	1.41	1.33
		Type	m	m	m	m	m	m	m
	**St**	L + S (%)	16.94	15.71	14.78	13.66	13.28	13.03	12.59
		L/S	1.15	1.11	1.97	1.64	1.14	1.06	1.15
		Type	m	m	sm	m	m	m	m
297876	**J^vs^**	L + S (%)	17.91	15.95	14.6	14.88	13.12	12.41	11.12
		L/S	1.05	1.66	1.85	1.8	1.32	1.27	1.71
		Type	m	m	sm	sm	m	m	sm
	**J^r^**	L + S (%)	16.22	16.2	15.31	13.69	12.71	12.54	13.32
		L/S	1.15	1.09	1.26	1.17	1.08	1.18	1.31
		Type	m	m	m	m	m	m	m
	**St**	L + S (%)	16.44	16.09	15.44	14.38	13.21	11.97	12.47
		L/S	1.19	1.52	1.36	1.36	1.44	1.1	1.13
		Type	m	m	m	m	m	m	m

**Table 3:** Chromosome arm ratio (L/S) and type of *Th. junceiforme*.

Species	Genome		Chromosome No.						
			1	2	3	4	5	6	7
414667	**J^vs^**	L + S (%)	17.89	16.33	15.15	12.73	12.82	13.24	11.84
		L/S	1.29	1.23	1.43	1.41	1.03	1.83	1.21
		Type	m	m	m	m	M	sm	m
	**J^r^**	L + S (%)	15.94	14.72	14.36	14.03	14.01	14.02	12.92
		L/S	1.36	1.25	1.56	1.15	2.11	1.61	1.4
		Type	m	m	m	m	sm	sm	m

**Table 4:** Chromosome arm ratio (L/S) and type of diploid material in the study.

Species		Chromosome No.						
		1	2	3	4	5	6	7
*Thinopyrum bessarabicum*	L + S (%)	16.58	15.12	14.92	14.21	13.82	12.93	12.42
	L/S	1.5	1.13	1.37	2.11	1.05	1.3	1.63
	Type	m	m	m	sm	m	m	m
*Th. elongatum*	L + S (%)	16.29	14.84	14.69	14.15	13.45	13.56	13.02
	L/S	1.23	1.1	2.06	1.61	1.36	1.17	1.15
	Type	m	m	sm	m	m	m	m
*Pseudoroegneria spicata*	L + S (%)	14.88	14.93	14.46	14.73	14.31	13.9	12.78
	L/S	1.51	1.04	1.11	1.39	1.07	1.42	1.13
	Type	m	m	M	m	m	m	m
*Dasypyrum villosum*	L + S (%)	16.24	15.79	14.48	14.13	13.57	13.48	12.32
	L/S	1.17	1.42	1.24	1.54	1.79	1.51	1.42
	Type	m	m	m	m	sm	m	m

**Table 5:** Karyotypes of IWG and its related species using different methods of evaluating karyotype asymmetry.

Type	Genome	ID					
		PI 228386	PI 297876	PI 414667	PI 340036	PI 531712	PI 563869
LC/SC	**J** **^vs^**	1.51	1.64	1.53	-	-	-
	**J** **^r^**	1.30	1.36	1.30	1.28	1.38	-
	**St**	1.45	1.42	-	-	-	1.26
AsK%	**J** **^vs^**	57.49%	59.60%	56.99%	-	-	-
	**J** **^r^**	54.84%	53.99%	59.29%	57.40%	58.32%	-
	**St**	56.17%	56.52%	-	-	-	55.09%
AI	**J** **^vs^**	1.73	2.06	1.56			
	**J^r^**	0.65	0.42	0.75	0.99	1.33	
	**St**	1.56	0.87				0.44

SC: the shortest chromosome length, LC: the longest chromosome length, LC/SC: ratio of longest/shortest chromosome, AsK%: The Karyotype asymmetry index (Arano, 1963 [43]), AI: karyotype asymmetry index (Paszko, 2006 [44]).

## Data Availability

All figures in this article are available for use without restrictions.

## Supplementary Materials

The following supporting information can be downloaded at: https://www.mdpi.com/article/10.3390/plants14020196/s1, Figure S1: Comparison of karyotype from the J^vs^ (A), J^r^ (B) and St (C) genomes.
