# Supplementary figures and images for: The Construction of a Standard Karyotype of Intermediate Wheatgrass and Its Potential Progenitor Species
Source: Plants (Basel). 2025 Jan 12;14(2):196. doi: 10.3390/plants14020196 (PMC11769444; doi:10.3390/plants14020196)

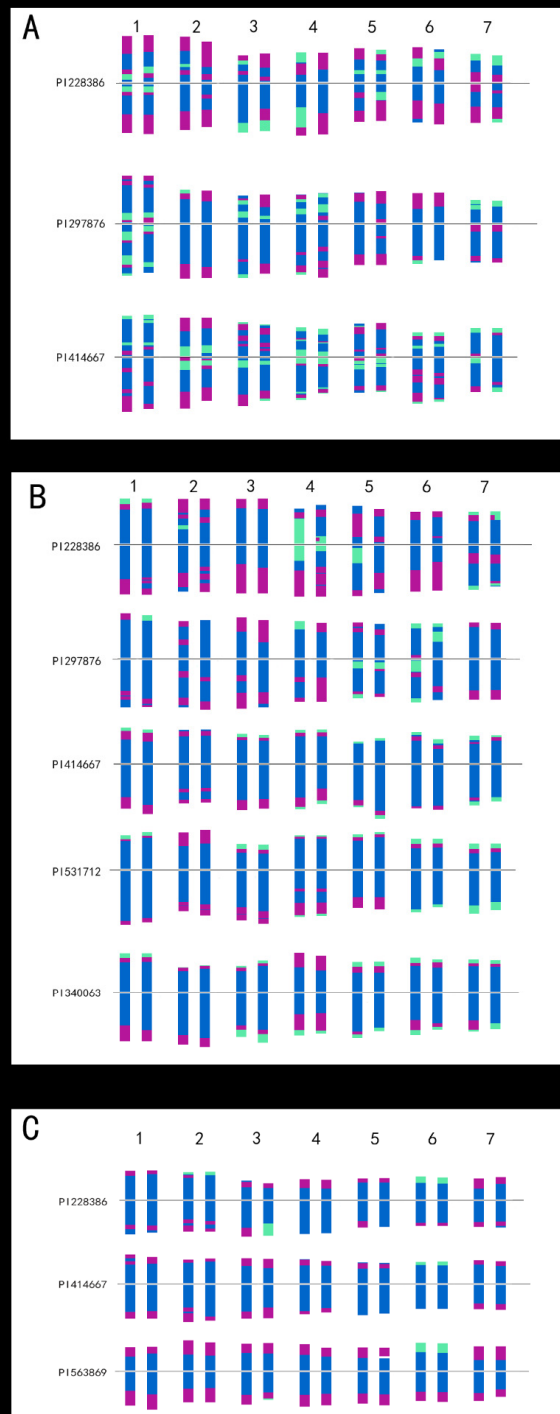

Supplemental Figure S1. Comparison of karyotype from the  $J^{vs}$  (A),  $J^r$  (B) and St (C) genomes.

Supplement: Supplementary file 1 [file plants-14-00196-s001.zip › plants-3393332-supplementary.pdf]
